# Exploring the Anticancer Potential of MonoHER (7-Mono-O-(β-Hydroxyethyl)-Rutoside): Mitochondrial-Dependent Apoptosis in HepG2 Cells

**DOI:** 10.3390/cimb47010036

**Published:** 2025-01-09

**Authors:** Chujie Li, Yue Wang, Jian Liang, Guido R. M. M. Haenen, Yonger Chen, Zhengwen Li, Ming Zhang, Ludwig J. Dubois

**Affiliations:** 1 Department of Pharmacology and Personalized Medicine, Research Institute for Nutrition and Translational Research in Metabolism (NUTRIM), Faculty of Health, Medicine and Life Sciences, Maastricht University, 6200MD Maastricht, The Netherlands; c.li@maastrichtuniversity.nl (C.L.); yue.wang@maastrichtuniversity.nl (Y.W.); g.haenen@maastrichtuniversity.nl (G.R.M.M.H.); 2 The M-Lab, Department of Precision Medicine, GROW—Research Institute for Oncology and Reproduction, Maastricht University, 6200MD Maastricht, The Netherlands; 3 Guangdong Provincial Key Laboratory of New Drug Development and Research of Chinese Medicine, Mathematical Engineering Academy of Chinese Medicine, Guangzhou University of Chinese Medicine, Guangzhou 510006, China; ljyyq@gzucm.edu.cn; 4 School of Pharmaceutical Sciences, Guangzhou University of Chinese Medicine, Guangzhou 510006, China; cye19961213@163.com; 5 School of Pharmacy, Chengdu University, 2025 Chengluo Avenue, Chengdu 610106, China; lizhengwen@cdu.edu.cn; 6 Hainan University-HSF/LWL Collaborative Innovation Laboratory, College of Food Sciences & Engineering, Hainan University, 58 People Road, Haikou 570228, China

**Keywords:** anticancer, apoptosis, flavonoids, MonoHER, mitochondrial dysfunction

## Abstract

Background/Aim: Flavonoids are a group of polyphenols, abundantly present in our diet. Although, based on their chemoprotective effects, intake of flavonoids is associated with a high anticancer potential as evidenced in in vitro and in vivo models, the molecular mechanism is still elusive. This study explores the antiproliferative and cytotoxic effects of the semi-synthetic flavonoid MonoHER (7-mono-O-(β-hydroxyethyl)-rutoside) in vitro on cancer cells. Materials and Methods: HepG2 liver, MCF7 breast, and H1299 lung cancer cells were grown under ambient conditions with or without MonoHER exposure. CCK8 assay was used to assess cell viability. Apoptosis, JC-1, and mitochondrial mass were determined using flow cytometry and confocal analysis. The effects of monoHER on apoptosis proteins were detected by confocal microscopy analysis and Western blot. Results: It was found that MonoHER can reduce HepG2 cells’ and MCF7 cells’ viability, but not H1299 cells’, and induced apoptosis only in HepG2 cells. MonoHER has the potential to enhance the expression of caspase-9 and caspase-3, to damage mitochondria, and to provoke the release of cytochrome C from the mitochondria. Conclusion: MonoHER can inhibit cell growth and induce apoptosis especially in HepG2 human liver cancer cells by triggering the mitochondrial signal transduction pathway, leading to the release of cytochrome C in the cytoplasm and the subsequent activation of caspase-9 and caspase-3. Future research should further explore MonoHER’s mechanism of action, efficacy, and potential for clinical translation.

## 1. Introduction

Cancer is one of the leading causes of morbidity and mortality worldwide, with an increasing burden due to lifestyle factors, aging populations, and environmental influences. According to the latest global cancer statistics from the World Health Organization (WHO), approximately 9.6 million people die from cancer each year, making it the second-leading cause of death globally. Among the various cancer types, liver cancer, breast cancer, and lung cancer are among the most prevalent and deadly worldwide, representing a substantial portion of cancer-related deaths and healthcare costs [1].

Distinctive characteristics of cancer cells include altered metabolism, disrupted cell cycle control, mutations, resistance to the immune response, and chronic inflammation [2]. The initiation of cancer involves various molecular processes, including oxidative stress, hypoxia, genetic mutations, and dysfunctional apoptotic control mechanisms [3]. A prevailing hypothesis suggests that cancer should be considered a metabolic disease influenced by mitochondrial dysfunctions and metabolic alterations [4]. Mitochondria, pivotal for cellular energy production, metabolic regulation, cell death signaling, and the generation of reactive oxygen species (ROS), play a central role in this hypothesis [5].

Mitochondrial dysfunction is evident in the metabolic abnormalities often observed in tumor cells, such as increased aerobic glycolysis, pH dysregulation, impaired lipid metabolism, and elevated ROS production [5]. Some of the consequences are that (i) the extracellular environment becomes acidic, promoting inflammation; (ii) glutamine-driven lipid biosynthesis increases, enhancing pathways involved in tumorigenesis and metas-tasis formation [2]; and (iii) cardiolipin levels in cell membranes are reduced, resulting in the compromised activity of enzymes, hyperpolarizing mitochondria, which correlates with the aggressiveness of cancer cells [6].

Phytotherapy is one of the potential options involving the usage of plants for the production of traditional drugs in the treatment of various cancers [7]. Nowadays, the application and evaluation of anticancer therapeutic effects of plants and their compounds are increasing. However, the mechanism by which these compounds act as anticancer agents is mostly unclear. As plants are good sources of antioxidants, the induction of antioxidant effects in the prevention and treatment of cancer is obvious [8]. One report has described anticancer and antioxidant activities of some algae such as Chlorophyta, Phaeophyta, and Rhodophyta. These algae comprise sources of polyphenols, such as flavonoids, isoflavones, cinnamic acid, benzoic acid, quercetins, etc. [9]. Most studies have suggested that the prevalence of cancer is lower in people consuming more fruits and vegetables that have antioxidative effects. Flavonoids, a group of dietary antioxidants, have demonstrated a wide range of biological effects with potential anticancer properties [10]. The flavonoid of interest is MonoHER (7-mono-O-(β-hydroxyethyl)-rutoside). MonoHER is chemically distinct from most flavonoids, which typically consist of a phenolic ring structure. Unlike common flavonoids such as quercetin, kaempferol, or catechins, which are polyphenolic compounds, MonoHER is a monoterpene glycoside, which could give it a unique ability. Previous research has suggested that MonoHER can protect healthy cells from the toxic effects of chemotherapeutics and may possess anticancer potential [11,12]. In this study, our aim was to further unveil the anticancer potential of MonoHER by exploring its possible mechanism of action, with a specific focus on the impact of MonoHER on the mitochondrial-driven apoptosis pathway in cancer cells.

## 2. Materials and Methods

### 2.1. Chemicals and Reagents

**Figure 1:**
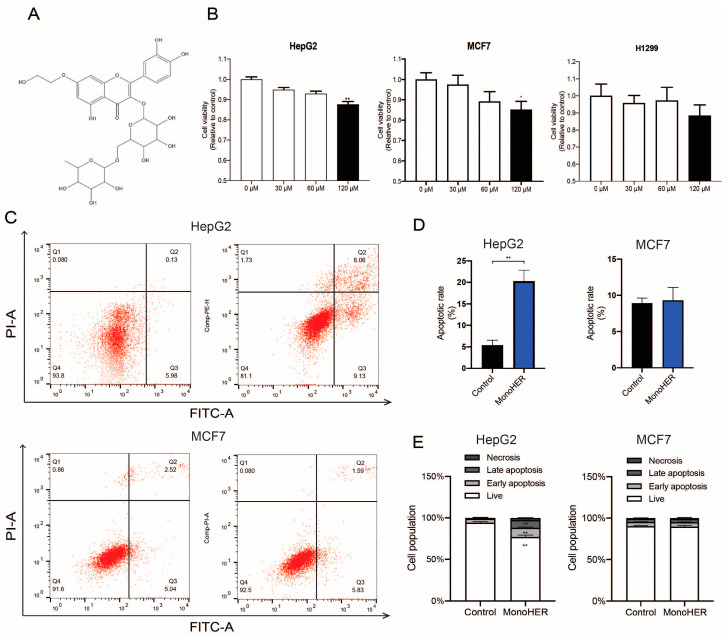
(**A**) The chemical structure of MonoHER. (**B**) Concentration (30 µM–120 µM)-dependent effect of MonoHER on cell viability. (**C**) Apoptosis induction by 120 µM MonoHER evaluated using Annexin-V/PI. Representative FACS images of untreated cells (control, left panel) and cells incubated with 120 µM MonoHER (right panel). Apoptosis rate (% of cells in 1 h) (**D**) and cell populations (%) (**E**) of untreated cells (control, left bar) and cells incubated with 120 µM MonoHER (right bar) from three independent experiments. The results are expressed as mean ± SEM. * *p* < 0.05, ** *p* < 0.01.

MonoHER (Figure 1A) was generously provided by Novartis Consumer Health, Nyon, Switzerland. It was dissolved in 36 mM NaOH in sterile water, resulting in a final concentration of 33 mg/mL (pH = 7.8–8) [11]. JC-1, Mito Tracker Green, Annexin V-FITC apoptosis assay kit for in situ apoptosis detection, RIPA, and PMSF were obtained from Beyotime Biotechnology (Shanghai, China). The BCA protein assay kit was purchased from Thermo Scientific. Antibodies against caspase 3 (ab13847), caspase 9 (ab32539), and cytochrome C (ab133504) were supplied by Abcam (Shanghai, China).

### 2.2. Cell Culture

The human HepG2 hepatocellular carcinoma was provided by the School of Pharmaceutical Sciences, Guangzhou University of Chinese Medicine, China, while the MCF7 luminal A breast adenocarcinoma and H1299 NSCLC adenocarcinoma cell lines were purchased from ATCC. Cells were cultured in DMEM supplemented with 10% (*v*/*v*) FBS, 100 U/mL penicillin, and 100 U/mL streptomycin, at 37 °C under a humidified incubator with 5% CO_2_. Cells (5 × 10^3^ cells per well) were grown in 96-well plates and incubated with various concentrations of MonoHER (final concentrations: 30 µM, 60 µM, and 120 µM) for 1 h.

### 2.3. Cell Viability Assay

Cell viability was assessed using the CCK8 assay kit (Beyotime Biotechnology, Shanghai, China) according to the protocol. After 1 h of MonoHER treatment, the medium was removed and replaced with fresh culture medium. Twenty-four h later, 10 µL CCK8 solution was added to each well and reaction was allowed for another two h at 37 °C [13] before assessing the absorbance of the sample at 450 nm with Multiskan Go (Thermo Scientific, Waltham, MA, USA), as a measure of cell viability. The cytotoxic doses of MonoHER were selected for further apoptosis assessments.

### 2.4. Apoptosis Evaluation by Annexin-V/PI

Cell apoptosis was detected using the Annexin V/PI detection kit according to the manufacturer’s instructions. Briefly, cells were washed twice with cold PBS, suspended in binding buffer, and stained with 5 µL annexin V-FITC and 10 µL PI for 15 min at 25 °C in the dark. Annexin V+/PI− cells were considered early-apoptotic cells, while Annexin V+/PI+ cells were considered late-apoptotic cells. Apoptosis analysis was conducted using an FACS Canto™ (BD Biosciences, San Jose, CA, USA) flow cytometer.

### 2.5. Immunofluorescence (IF) Staining

To detect caspase activity, cells were fixed with 4% paraformaldehyde after treatment and permeabilized with Triton X-100 (0.1%) for 10 min. The cells were then sealed with 5% BSA for 30 min, washed with PBS, and incubated with different antibodies, such as anti-caspase-3 and anti-caspase-9, overnight at 4 °C. Subsequently, the cells were incubated with Alexa Fluor 488-conjugated IgG antibody for 2 h at room temperature and washed with PBS. Finally, the cells were stained with DAPI for 5 min at room temperature and were imaged and analyzed using laser confocal microscopy (LSM 800, ZEISS, Shanghai, China).

### 2.6. Measurement of Mitochondrial Mass

To detect mitochondrial mass, cells were stained with MitoTracker Green (Beyotime Biotechnology, Shanghai, China) at a final concentration of 100 nM for 30 min after MonoHER treatment. The mass of the mitochondria was determined by laser confocal microscopy (LSM 800, ZEISS).

### 2.7. Mitochondria Membrane Potential (ΔψM) Determination

The mitochondrial membrane potential was determined via JC-1 kit. Treated cells were incubated with JC-1 solution at 37 °C for 20 min and were detected using an FACS Canto™ flow cytometer. Data were analyzed using Flowjo 10.8.1 software.

### 2.8. Western Blotting Analysis

To detect the release of cytochrome C from mitochondria, mitochondria and cytosol were isolated, and cytochrome C in the cytosol was detected by Western blot. Isolation of mitochondrial and cytosolic proteins was performed using the Mitochondria/cytosol Fractionation Kit (Beyotime, Shanghai, China). The protein concentration was determined using the BCA kit. Proteins were mixed with loading buffer and boiled for 5 min. Equivalent proteins were electrophoretically separated using SDS–polyacrylamide gels and transferred onto PVDF membranes. The membranes were blocked with 5% BSA for 2 h, followed by incubation overnight at 4 °C with primary antibodies, such as anti-cytochrome C (1:500) and anti-GAPDH (1:2000). The next day, the membranes were incubated with HRP-conjugated secondary antibodies for 2 h at room temperature. After washing bands three times with PBST, bands were detected by ECL^TM^ Prime Western Blotting system (Cytiva, GERPN2232, Marlborough, MA, USA), and the intensities of bands were quantified using Image J 1.50i gel analysis software.

### 2.9. Statistical Analysis

Statistical analysis was performed using SPSS software (version 18.0; SPSS, Chicago, IL, USA). All results are expressed as mean ± SEM. The results were evaluated using one-way ANOVA, Student’s *t*-test, and further compared with Tukey’s multiple analysis when appropriate. Values of *p* < 0.05 were considered statistically significant.

## 3. Results

### 3.1. MonoHER Caused Cell Death in HepG2 Cells and MCF7 Cells

To assess the cytotoxic effect of MonoHER on different cell lines, CCK-8 tests were performed. As depicted in Figure 1B, monoHER treatment reduced the viability of the HepG2 and MCF7 cells in a dose-dependent manner. At a concentration of 120 µmol/L, MonoHER caused a notable cytotoxic effect in the HepG2 cells (*p* < 0.01) and MCF7 cells (*p* < 0.05), but not in the H1299 (*p* = 0.604) cells.

### 3.2. MonoHER Induced Apoptosis Only in HepG2 Cells

Flow cytometric analysis revealed that MonoHER at a concentration of 120 µM induced apoptosis in the HepG2 cells. MonoHER treatment significantly (*p* < 0.01) increased the percentage of apoptotic cells (Q2 + Q3), from 6.69% (control) to 21.9% (120 µM MonoHER), confirming that MonoHER induces apoptotic cell death in HepG2 cells, but not in MCF7 cells, as there is no significant difference between the control and monoHER treatment groups in the apoptotic rate (Figure 1C–E).

### 3.3. MonoHER Caused Mitochondrial Damage in HepG2 Cells

**Figure 2:**
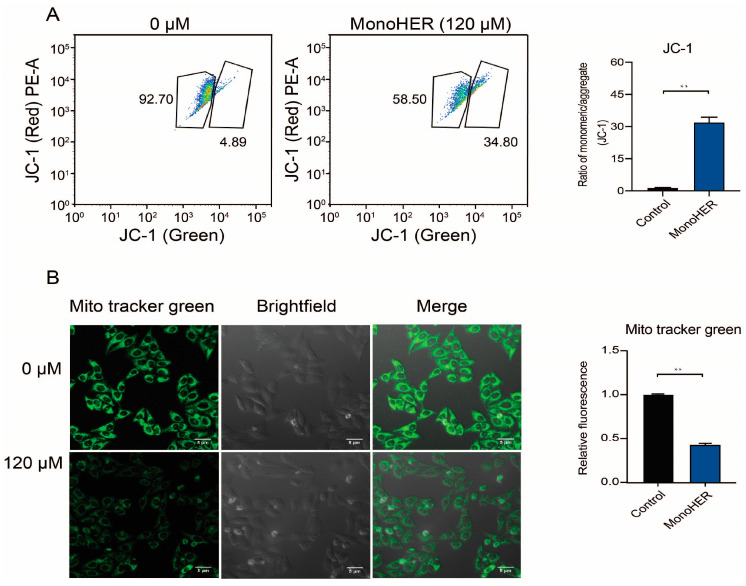
Effects of 120 µM MonoHER on mitochondrial dysfunction in HepG2 cells. (**A**) Mitochondrial membrane potential (ΔψM) was measured by JC-1 staining, followed by flow cytometry. (**B**) The mass of mitochondria was measured using Mito Tracker Green. The fluorescence intensity was calculated using Image J. Mean ± SEM from three independent experiments is shown. ** *p* < 0.01.

Mitochondria play a vital role in apoptosis triggered by many stimuli. To investigate whether the functional integrity of mitochondria was preserved after treatment with MonoHER, JC-1 staining was performed. Treatment of HepG2 cells with a low mitochondrial membrane potential with 120 µM MonoHER resulted in a significantly (*p* < 0.01) higher ratio of green fluorescence (JC-1 monomers) to red fluorescence (JC-1 aggregates) compared to the control group (Figure 2A), indicative of a lowered mitochondrial membrane potential and therefore mitochondrial damage.

To determine whether mitochondrial alterations occurred during MonoHER-induced apoptotic cell death, we examined mitochondrial mass alterations. As shown in Figure 2B, in the 120 µM MonoHER-treated cells, the green fluorescence intensity significantly (*p* < 0.01) decreased compared to the control group, indicating that 120 µM MonoHER decreases the mitochondrial mass.

### 3.4. MonoHER Triggered Mitochondria-Dependent Apoptosis in HepG2 Cells

**Figure 3:**
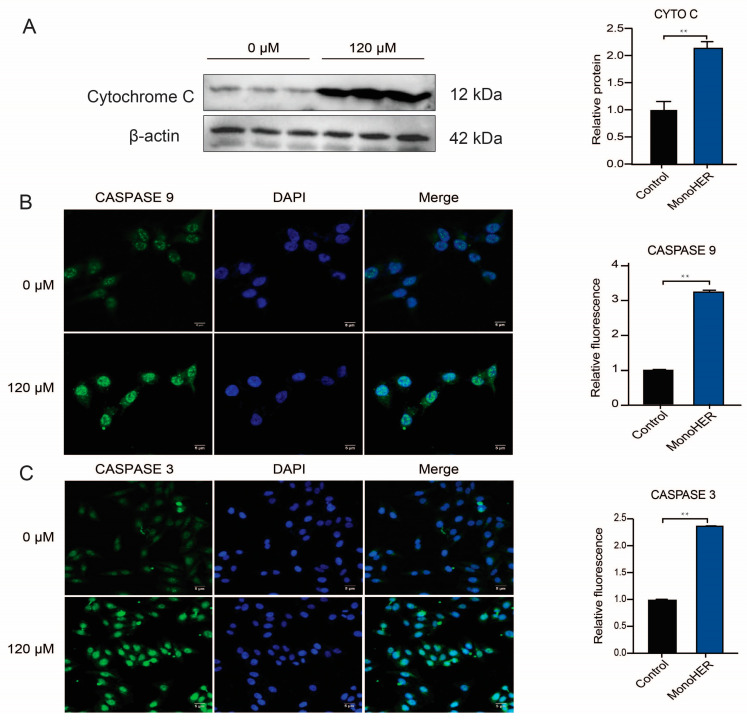
Western blotting was performed to assess the effects of MonoHER on the expression of cytochrome C released from mitochondria (**A**) and immunofluorescence staining was used to detect the expression of caspase 9 (**B**) and caspase 3 (**C**). The scale bar in each panel represents 5 µm. Mean ± SEM from three independent experiments is shown. ** *p* < 0.01.

Western blotting and immunofluorescence data revealed that the 120 µM MonoHER treatment significantly upregulated caspase-3 (*p* < 0.01), caspase-9 (*p* < 0.01), and cytochrome C (*p* < 0.01) release from mitochondria (Figure 3), confirming the occurrence of mitochondria-dependent intrinsic apoptosis in HepG2 cells treated with MonoHER (Figure 3).

## 4. Discussion

Considerable research has focused on the prevention and therapy of cancer by flavonoids [14]. Recent studies have highlighted the antiproliferative properties of flavonoids against various cancer cells [15], mediated through various mechanisms, including the regulation of oncogene and tumor suppressor gene expression [16], the inhibition of signaling pathways involving MAPK, NF-κB, Nrf, and AP-1, and the induction of cell cycle arrest and apoptosis [17]. Factors involved include p53, Bcl-2, and the caspase family [18].

In this study, our focus was on the flavonoid MonoHER. MonoHER possesses potent redox-modulating activity and has the ability to chelate transition metals, such as iron [19]. These properties may explain its previously observed high efficacy in protecting healthy cells [20]. In our previous study, we found that MonoHER can enhance the antitumor activity of doxorubicin in soft tissue sarcoma patients [21,22]. In this in vitro study, we aimed to assess the anticancer potential of MonoHER and its underlying mechanism on cancer cells. According to our previous research, 50 µM MonoHER can sensitize WLS-160 cells to doxorubin-induced apoptosis [22]. In this study, we chose 30 µM, 60 µM, and 120 µM as the experimental concentrations. The results of this study demonstrate, for the first time, that MonoHER exhibits potential antiproliferative and cytotoxic effects, inducing apoptosis in HepG2 cells, but not in MCF7 cells or H1299 cells. The possible reasons why monoHER does not work effectively in these cell lines could be the following: (1) hepatocytes are specialized for metabolic processes, including handling polyphenolic compounds like flavonoids. These cells therefore have a higher expression of transporters and enzymes involved in flavonoid uptake and metabolism, such as phase I (cytochrome P450) and phase II (glucuronidation, sulfation) enzymes. This could explain why liver cancer cells are more efficient at processing flavonoids into active metabolites capable of inducing apoptosis than other cells [23]; (2) the differential sensitivity of flavonoids across various cell types could also be a factor. The anticancer effects may become apparent in breast cancer and lung cancer cells when the concentration of monoHER is increased; and (3) several studies have shown that the absorption, distribution, and cellular uptake of flavonoids are heavily influenced by membrane transporters, such as ATP-binding cassette (ABC) transporters and solute carrier (SLC) transporters. Different cell types express varying levels of these transporters, which can affect the intracellular concentration of flavonoids and, consequently, their biological effects [24,25].

Chemotherapeutic agents are known to induce tumor regression through apoptosis [26]. In our study, the results from annexin V/PI co-staining clearly demonstrate that 120 µM MonoHER induces apoptosis in HepG2 cells. Confocal and flow cytometry results also show that 120 µM MonoHER decreases the mitochondrial mass and mitochondrial membrane potential. This suggests that MonoHER can cause apoptosis in HepG2 cells, by disrupting mitochondria, and consequently releasing cytochrome C, which, due to mitochondrial dynamics, affects mitochondrial function. This finding is further confirmed by the upregulated caspase cascade occurring during apoptosis.

**Figure 4:**
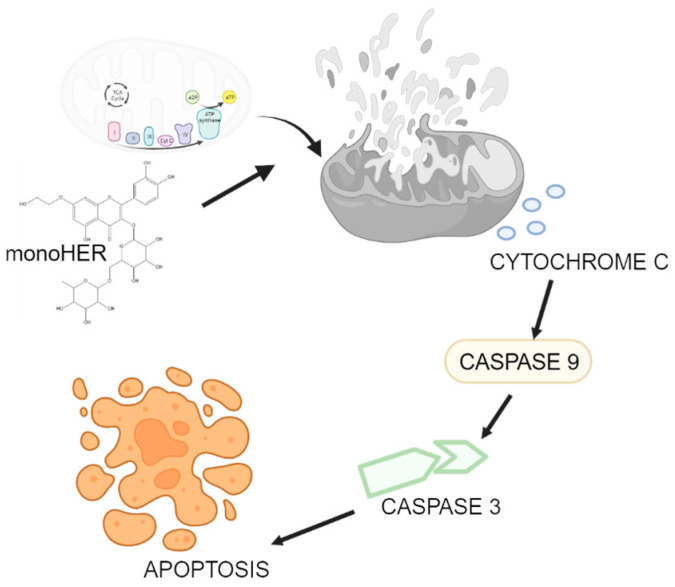
The mechanism of MonoHER-induced mitochondrial-dependent apoptosis in HepG2 cells. Figure 4 was created with biorender.

Apoptosis is induced by a series of caspase activations, leading to cell death [27]. Caspases exist as inactive procaspases and are cleaved into their active forms [28]. In our study, MonoHER induced the formation of cleaved forms of caspase-9 and -3. Apoptosis can be divided into extrinsic and intrinsic pathways. The extrinsic pathway involves the activation of caspase-8, while the intrinsic pathway involves mitochondrial-dependent apoptosis, leading to the activation of caspase-9, followed by cytochrome C release from mitochondria [29]. Caspase-9 is an initiator caspase that activates downstream effector caspases, such as caspase-3, which systematically dismantles cells [30,31]. Our data show that MonoHER induces apoptosis in HepG2 cells through the activation of caspase-3, caspase-9, and cytochrome C in an intrinsic manner, as depicted in Figure 4.

## 5. Limitation

Our study comprises in vitro experiments using cancer cell lines. While in vitro models offer controlled conditions for mechanistic investigations, the complex and dynamic in vivo microenvironment cannot be mimicked in vitro and may yield different results. Therefore, translating in vitro findings to in vivo systems presents significant challenges. Additionally, the concentration of MonoHER required to elicit the observed effects, notably 120 µM, is relatively high and may not be achievable in a physiological context. However, the incubation time of one hour is short compared to the retention time of MonoHER in vivo after a single administration that in some cells can exceed one day [31,32], indicating that in vivo lower concentrations than 120 µM might induce apoptosis. Moreover, the relevance of other proposed anticancer pathways, such as the prevention of carcinogenesis, and the synergetic potentiation of MonoHER of other anticancer mechanisms should be taken into consideration. Finally, future studies should aim to confirm the observed effects of MonoHER in animal models and in human tissues, to better understand its pharmacokinetics, bioavailability, and therapeutic potential in a whole organism context.

## 6. Conclusions

In conclusion, the results of this study clearly demonstrate that MonoHER can inhibit cell growth and induce apoptosis especially in HepG2 human liver cancer cells. It triggers the mitochondrial signal transduction pathway, leading to the release of cytochrome C in the cytoplasm and the activation of caspase-9 and caspase-3. This study has provided new insights into the effect of MonoHER and calls for follow-up research on the anticarcinogenic potential of this flavonoid, especially relevant for liver cancer, as it has a tremendously high mortality rate.

## Data Availability

No data were used for the research described in the article.
